# Supplementary material for: Sorting at embryonic boundaries requires high heterotypic interfacial tension
Source: Nat Commun. 2017 Jul 31;8:157. doi: 10.1038/s41467-017-00146-x (PMC5537356; doi:10.1038/s41467-017-00146-x)
Supplement: Supplementary file 2 — Supplementary Software 1 [file 41467_2017_146_MOESM2_ESM.zip › PottsModel/SrcPottsModel/doc/index-files/index-5.html]

E-Index


JavaScript is disabled on your browser.


Skip navigation links


- Overview
- Package
- Class
- Use
- Tree
- Deprecated
- Index
- Help

- Prev Letter
- Next Letter

- Frames
- No Frames

- All Classes

A B C D E F G H I J K L M N O P R S T U V W 


## E

EndoSimulation - Class in simulations
:   A simple simulations using the common-properties configuration.

EndoSimulation() - Constructor for class simulations.EndoSimulation


EnergyFunction - Interface in model


EnergyFunction.Energy - Enum in model


EnergyManager - Class in model
:   Manages energy functions.

EnergyManager(Constants, Constants.Model, PottsEngine.StateVariables) - Constructor for class model.EnergyManager


EnergyStatistic - Class in engine


EnergyStatistic(PottsEngine) - Constructor for class engine.EnergyStatistic


EnergyStatistic(PottsEngine, int) - Constructor for class engine.EnergyStatistic


EnergyTracker(List<EnergyFunction.Energy>) - Constructor for class engine.Utils.EnergyTracker


engine - package engine


EngineObserverPanel - Interface in gui


equals(Cell) - Method in class model.Cell


equals(Object) - Method in class model.Pixel

A B C D E F G H I J K L M N O P R S T U V W

Skip navigation links


- Overview
- Package
- Class
- Use
- Tree
- Deprecated
- Index
- Help

- Prev Letter
- Next Letter

- Frames
- No Frames

- All Classes
